# Caroli's Syndrome: A Case Report and Literature Review

**DOI:** 10.7759/cureus.50871

**Published:** 2023-12-20

**Authors:** Muhammad Nabeel Shafqat, Muhammad Yousuf Y Memon, Salman Javed, Sai Gautham Kanagala, Momina Saleem

**Affiliations:** 1 Department of Gastroenterology and Hepatology, Allied Teaching Hospital Gujranwala, Gujranwala, PAK; 2 Department of Gastroenterology, King Saud Hospital, Unaizah, SAU; 3 Department of Gastroenterology and Hepatology, Services Institute of Medical Sciences, Lahore, PAK; 4 Department of Internal Medicine, Osmania Medical College, Hyderabad, IND; 5 Department of Medicine, Allied Teaching Hospital Gujranwala, Gujranwala, PAK

**Keywords:** intra-hepatic cysts, portal hypertension, congenital hepatic fibrosis, caroli's disease, caroli’s syndrome

## Abstract

Synonymous with congenital non-obstructive saccular or fusiform intra-hepatic duct dilatation and congenital communicating cavernous ectasia of the intra-hepatic biliary tract, Caroli’s syndrome (CS) is an extremely rare fibro-polycystic liver disorder characterized by ductal plate malformation and consequent peri-portal fibrosis due to segmental intra-hepatic duct dilatation. No more than 200 cases of the syndrome have been reported since 1958. CS may affect one or both lobes of the liver, but more commonly it affects the left hepatic lobe. We describe a rare case of CS localized to the right hepatic lobe in a 21-year-old male, who presented with complaints of upper gastrointestinal (GI) bleeding without any signs or stigmata of chronic liver disease. Personal as well as family history was non-significant except positive for consanguineous parental marriage. General physical examination was unremarkable except for pallor, and upper GI endoscopy revealed columns of bandable esophageal varices which led us to a line of investigations to identify the cause of portal hypertension. Blood tests were non-specific, though imaging studies chiefly abdominal ultrasound, CT abdomen and pelvis with contrast, and magnetic resonance cholangiopancreatography (MRCP) led us to confirmation of the diagnosis of CS in the right hepatic lobe with manifestations of portal hypertension as the predominant feature. Diagnosis was confirmed on liver biopsy which showed right-sided cystic dilations with congenital hepatic fibrosis.

## Introduction

First described in 1958 by Dr. Jacques Caroli, a French gastroenterologist, Caroli's syndrome (CS) is synonymous with congenital intra-hepatic bile duct dilatation, congenital non-obstructive saccular or fusiform dilatation of the intra-hepatic bile ducts, and congenital communicating cavernous ectasia of the intra-hepatic biliary tract [1,2]. Caroli’s disease (CD) has two types: type I (simple CD), which is pure cystic intra-hepatic bile duct dilatations, and type II (complex CD or Caroli syndrome), which is hepatic fibrosis, cirrhosis, and portal hypertension [2].

CS is a rare congenital disorder characterized by segmental dilatation of the intra-hepatic ducts and hepatic fibrosis. It is estimated that CS is present in 1/1,000,000 of the population [2]. Though the etiology and pathophysiology are still not very well known, CS is inherited in an autosomal recessive pattern. It is associated with genetic mutations in PKHD1 gene, which encodes a protein fibrocystin that helps build bile ducts and involves ductal plate malformation and consequent peri-portal fibrosis [1,3,4].

As opposed to CD, which involves congenital hepatic impairment confined to the development of cysts and mostly presents as right hypochondrial pain, obstructive jaundice, and cholangitis, CS is a cystic disease is associated with congenital or primary hepatic fibrosis, so the presentation is mostly the result of portal hypertension or hepatic insufficiency, manifested as ascites, splenomegaly, peripheral edema, esophageal varices, and coagulation disorders [5]. CS may involve the biliary tract either in a focal or a multifocal manner. It may involve the entire liver, a lobe, or even a single segment. CS more commonly affects the left hepatic lobe and localized right hepatic lobe involvement is rare. At present, it is considered a part of group V of the Todani classification of biliary tract cystic diseases [2,5].

There are no pathognomonic signs or symptoms associated with CS. Affecting both genders equally, with a male-to-female ratio in both CD and CS of 1:1.8, the clinical manifestations may be insidious, and patients mainly present in one of the following two ways: ‘intra-hepatic ductal ectasia and stagnation of bile’ (recurrent cholangitis and/or choledocholithiasis) or ‘portal hypertension’ (gastrointestinal (GI) bleed, splenomegaly, ascites) [3,5]. Physical examination findings include either no findings or hepatosplenomegaly, peripheral edema, ascites, splenomegaly, hepatomegaly, hepatic insufficiency, and/or portal hypertension. Laboratory findings are mostly non-specific [6].

CS is also associated with pancreatic cysts, cavernomatous portal vein transformation, choledochal cysts, renal tubular ectasias, cortical cysts, renal medullary spongiosis, medullary cystic disease, autosomal recessive polycystic kidney disease (ARPKD), or even autosomal dominant polycystic kidney disease (ADPKD), nephrolithiasis, hypertension, or pyelonephritis in infants and an increased risk of cholangiocarcinoma [3,5,6]. The risk of cholangiocarcinoma is 2.5-17.5% in CS, 100 times higher than in patients with normal hepatobiliary ducts and 10 times higher than in those with calculi [7]. It is also characterized by intraductal calculi formation and increased susceptibility to infection [2].

While generally diagnosed within the first 20 years of life, CS may also remain asymptomatic for the entire life of the individual, or it can be diagnosed as late as the fifth decade [4,5]. A definitive diagnosis can be established by histopathology, but the first-line diagnostic investigation methods used because of their convenience and non-invasiveness are ultrasonography (USG), computed tomography (CT), and magnetic resonance imaging (MRI) [3].

The first step to timely intervention, complication control, and surveillance of CS is an early diagnosis [3]. Recurrent episodes of cholangitis are usually an indication of hemi-hepatectomy i.e. surgical resection of the part of the liver where the ducts are too wide [1,8,9].

No more than 200 cases have been reported in the literature since Caroli’s paper in 1958 [4]. Hence, we present a case of a male adult with CS. This case report aims to add a rare presentation of CS to the existing knowledge of this extremely rare congenital disorder.

## Case presentation

We present a rare case of a 21-year-old male, a university student, with no known co-morbidities, who was in a perfect state of health when he experienced an episode of hematemesis on day 1. He consulted a local general physician who managed him conservatively based on a provisional diagnosis of Mallory-Weiss tears, and he was sent home. On day 2, around 4 to 6 months later, the patient started experiencing epigastric burning, generalized body weakness, and weight loss, and later on he suffered from multiple episodes of melena, and was asked to consult a gastroenterologist. The patient denied any previous episodes of melena. He also denied a history of alcohol intake or smoking. The patient had no positive family history of liver disease, and the only notable point in his history was his parents’ consanguineous marriage. On physical examination, the patient did not have any stigmata of chronic liver disease except for pallor i.e. there was no jaundice, abdominal tenderness, dilated veins, ascites, or visceromegaly. His blood pressure was 110/70 mmHg and his heart rate was 106/min. The remaining cardiovascular, neurological, musculoskeletal, and respiratory examinations were unremarkable. Esophago-gastro-duodenoscopy (EGD) was planned keeping in view his history. Preliminary reports revealed severe anemia with a Hb level of 3.6 g/dl and it increased up to 9.5g/dl after transfusion of six red blood cell packs. After stabilizing the patient, EGD was performed which revealed multiple large bandable varices, and esophageal variceal band ligation (EVBL) was done. This called for further investigations to look for the cause of portal hypertension, and a number of investigations were carried out. Laboratory data of peripheral blood samples are shown in Table 1. CBC showed a Hb level of 3.6 g/dL, WBC 3.09x10^3^/uL, and platelets 80x10^3^/uL; viral markers (HbsAg and Anti-HCV) were negative, iron indices, urinary copper and ceruloplasmin levels were all within normal range, ophthalmology consult for slit lamp examination revealed no Kayser-Fleischer rings; blood work showed normal renal function tests and serum electrolytes, ruling out most common causes of cirrhosis.

**Table 1:** Laboratory data of peripheral blood smear at presentation and then at follow-up at six months and 12 months. CA-19-9: Carbohydrate antigen 19-9; CEA: carcinoembryonic antigen

Laboratory parameters	Results at presentation	Results at six months	Results at 12 months
Hemoglobin (12 - 16 g/dl)	3.6	8.9	11.2
Leukocytes (4.5 - 11 x 10^9^/L)	3.09	4.12	3.74
Platelet (150,000 - 450,000 x uL)	80	110	98
International normalized ratio	1.04	1.4	1.02
Prothrombin time (10 - 13 sec)	13.01	18.2	14.1
Glycemia (70 - 100 mg/dl)	96	89	94
Urea (5 - 20 mg/dl)	30	33	36
Blood Urea Nitrogen (6 - 24 mg/dl)	14	13	16
Creatinine (0.7 - 1.3 mg/dl)	1.02	0.99	1.03
Lactate Dehydrogenase (140 - 280 U/L)	163	148	156
Alkaline phosphate (44 - 147 IU/L)	130	142	136
Alanine Aminotransferase (8 - 33 U/L)	45	67	34
Aspartate Aminotransferase (7 - 55 U/L)	35	87	33
Albumin (3.4 - 5.4 g/dl)	4.1	3.8	3.4
Bilirubin (0.1 - 1.2 mg/dl)	0.6	0.6	0.8
Alpha-fetoprotein (4 - 10 ng/ml)	6.04	-	9.42
CA 19-9 (0 - 37U/ml)	53.46	-	38.91
CEA (0 - 2.5 ng/ml)	1.05	-	2.01

**Figure 1:**
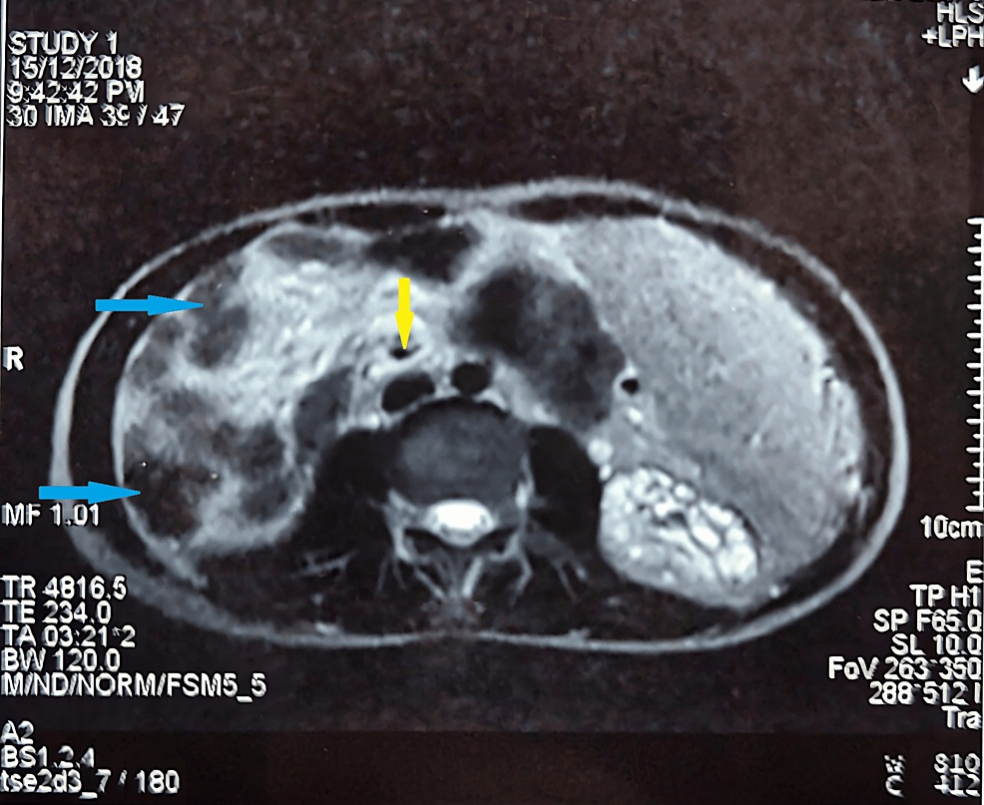
Magnetic resonance imaging showing hepatomegaly with cystic dilatations (blue arrows) and biliary ectasia (yellow arrow).

Abdominal USG showed splenomegaly, a 5.4x3.2 cm hepatic cyst in segment VII, partial thrombosis of the right hepatic vein, and bilateral renal parenchymal disease grade III. On USG, liver cysts were showing a central dot sign confirming the possibility of segmental dilated intra-hepatic biliary channels. This was followed by magnetic resonance imaging, which showed mild hepatomegaly with smooth marginal surfaces, cystic dilatations of intra-hepatic bile ducts predominantly in the right lobe, and ectasia of the biliary channels in both lobes and splenomegaly (Figure 1).

**Figure 2:**
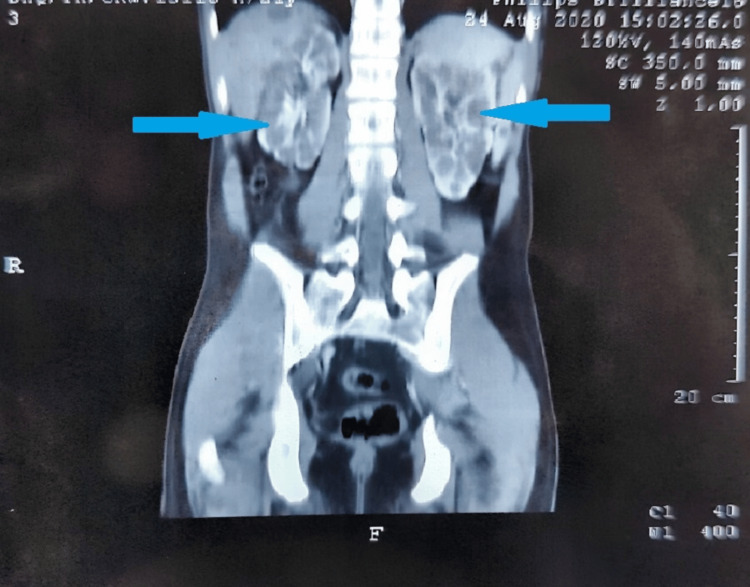
Contrast-enhanced computed scan showing bilaterally enlarged kidneys with multiple small parenchymal cysts (blue arrows).

A contrast-enhanced CT scan of the patient also demonstrated bilaterally enlarged kidneys with multiple small parenchymal cysts, all pointing toward a diagnosis of CS (Figure 2).

**Figure 3:**
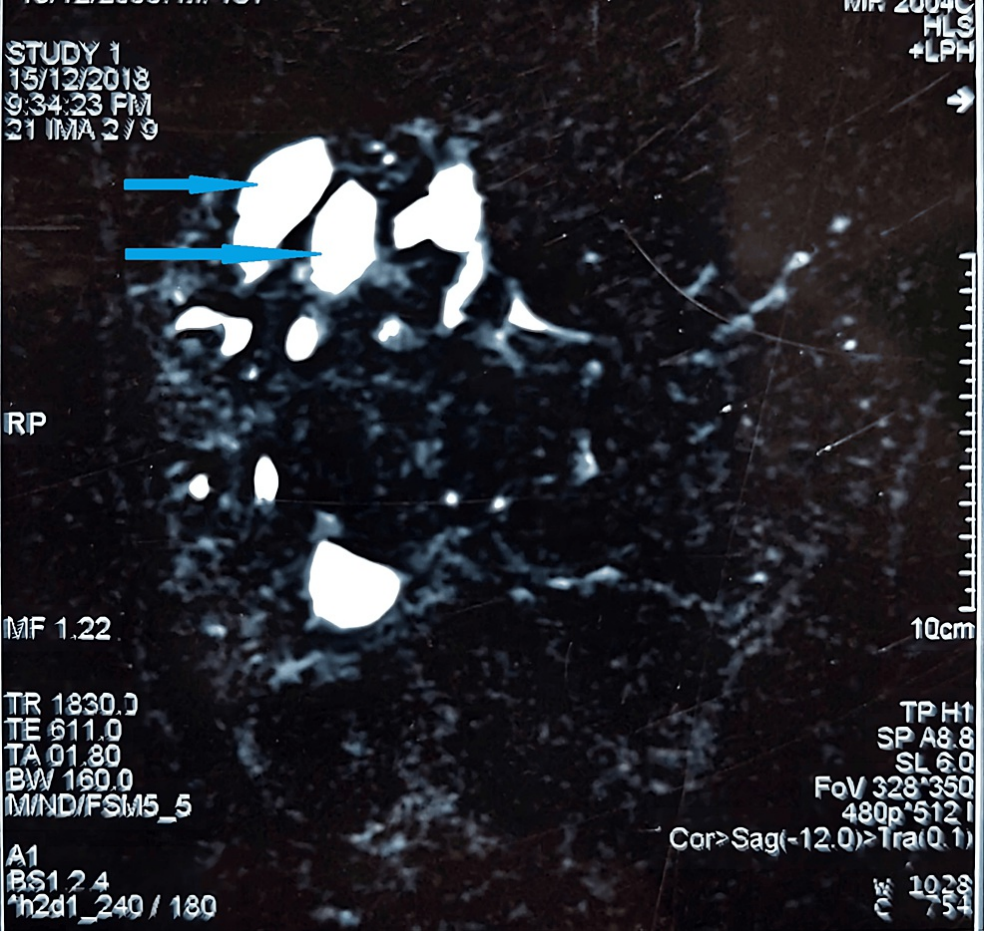
Magnetic resonance cholangiopancreatography (MRCP) demonstrating segmental intra-hepatic cysts (blue arrows).

Magnetic resonance cholangiopancreatography (MRCP) showed markedly dilated segmental bile ducts in the right lobe of the liver, particularly in segments 8, 5, and 6, mild dilatation of left-sided ducts, large dilated cystic spaces, significantly enlarged spleen, and a few small cysts in kidneys (Figure 3).

A confirmatory USG-guided biopsy of the liver from the left lobe was carried out one month later to assess cirrhosis and confirm the diagnosis of CS. Since the diagnosis, the patient has presented multiple times with UGIB, and, on each occasion, EVBL was performed to control bleeding. His most recent EGD showed three columns of small bandable esophageal varices with red signs, previously sclerosed varices, and severe portal hypertensive gastropathy with a small bunch of fundal varix without any stigmata of recent bleeding. The patient has been counseled about liver transplant options but due to poor resources and the non-availability of a donor liver, the patient has been constantly refusing the transplant. The patient has been on symptomatic treatment with omeprazole and carvedilol and regular endoscopic follow-up. However, now, due to progression of his disease and poor control, he is strongly advised to undergo a liver transplant. The patient is under regular follow-up with a gastroenterologist and a nephrologist to avoid any further complications.

## Discussion

CS is an autosomal recessive disorder and recessive genetic disorders are the result of an individual inheriting one defective gene from each parent. An individual with one working and one defective gene will be a carrier for the disease and will not develop symptoms. Consanguineous marriages, as in this patient’s history, have increased chances of bearing children with a recessive condition, as in our case [1,10,11].

Inaugural symptoms usually begin between adolescence and early adulthood, as in our case, but can appear at any time. A smaller percentage of patients may remain asymptomatic throughout life and be diagnosed accidentally by imaging [5,12]. The most frequent symptoms in decreasing order are right upper quadrant pain, fever, anorexia, variceal bleeding, lethargy, and jaundice in both CS and CD [13]. However, in this patient, the presenting complaint was hematemesis and melena and surprisingly, he had never had right upper quadrant pain, fever, anorexia, or jaundice. The chief clinical presentation is hematemesis/melena/hematochezia if portal hypertension is predominant [5,6,14].

Patients who present with CD symptoms before 40 years of age are more likely to have type II CD (or CS), as in our case, with no gender predominance observed. Patients may also present with non-specific symptoms such as fatigue and anorexia, usually due to severe infections such as septicemia or abscesses, but our patient had none of these. Calculi, if formed, aggravate bile obstruction and lead to biliary cirrhosis [1].

USG is the best initial examination because it is cheap, fast, and non-invasive, though it has low specificity and is operator-sensitive. It may show irregular dilatation of the intra-hepatic bile ducts, sometimes associated with extrahepatic ductal dilatation owing to cholelithiasis [5,15]. USG findings have an accuracy of 27.3% in CD or CS and include intra-hepatic cystic anechoic areas, consisting of fibrovascular bundles (comprising hepatic arteries and portal veins, clearly shown in Doppler USG), linear bridging or septations, and stones. It does not distinguish CD cysts from cysts due to other conditions, e.g. polycystic liver disease [2]. Evidence of portal hypertension can be assessed in Doppler studies [8]. The dilated biliary channels appear anechoic on USG and hypodense on CT [6,15].

CT findings are 71.4% accurate and may show central dots (the central dot sign - a small focus of strong contrast enhancement within the dilated intra-hepatic ducts; highly specific for CS), representing a specific and essential sign, indicating fibrovascular bundles comprising the portal vein radical and a hepatic artery branch bridging the saccule [16]. CT is best for evaluation of extra-hepatic manifestations of CS which may not be delineated by USG, such as pancreatitis, pancreatic pseudocyst, phlegmon, subhepatic or subphrenic abscesses so should be done in every case of suspected CS [3,4,6,17]. MRIs have an accuracy of 77.8% as per studies, so they do not hold more advantages over CT [2].

Besides non-invasive imaging techniques such as USG, CT, and MRI, sometimes, due to diagnostic uncertainty, ERCP and MRCP are needed to confirm the diagnosis [1,3,16]. ERCP has the highest sensitivity, as it can view the entire biliary tree and also identify intra-hepatic masses, if any. Cystobiliary communication characteristic of CD/CS can only be directly demonstrated by ERCP, percutaneous transhepatic cholangiography, or intra-operative cholangiography. However, it is associated with a risk of infection and acute pancreatitis because of its invasiveness, so it is not needed when the diagnosis of CS or CD has already been established [2,4].

MRCP in CD shows what is named the “lollipop tree” aspect (T2 and most notably T1 sequences after injection of contrast), which appear as diverticulum-like saccules of intra-hepatic bile duct dilatations of variable shapes, sizes, and distribution, communicating freely with the bile duct [16]. However in CS, the cystic bile duct dilatation is usually smaller (<2 cm) and periportal hepatic fibrosis is observed on T2-weighted sequences as high-signal regions among the portal veins [6,18]. It also helps rule out other diseases such as polycystic liver disease and multiple liver abscesses. The communication between the bile duct and the saccule differentiates CD from polycystic liver disease, where the bile duct does not communicate with the saccule. Also, the saccular dilatations in CS contain bile, while the cysts in polycystic liver disease do not contain bile [2,4,8,17]. It has a high specificity and sensitivity besides being less invasive, and is at present, the investigation of choice. Despite being expensive it is universally accepted as the most consistent method for assessment of disease severity and extent [5].

Liver biopsy followed by histopathological examination is definitive and shows dense hepatic and portal fibrosis, dilated bile ducts, and secondary cirrhosis. Being invasive, liver biopsy is done in case of diagnostic uncertainty and it was performed on our patient to confirm the diagnosis [2,6,12].

Differential diagnoses of CS/CD include polycystic liver disease, primary sclerosing cholangitis, von Meyenburg complex, and choledochal cysts. von Meyenburg complex is characterized by multiple small cystic nodules (<1.5 cm) on MRCP that do not communicate with the biliary tree [6].

Management involves multi-disciplinary care by a team of gastroenterologists, hepatologists, and transplant hepatologists [1,18]. In clinical and socioeconomic settings like ours with limited resources, the treatment is chiefly supportive and conservative. The aim of treatment is to prevent and treat biliary tree infections and portal hypertension complications and prevent morbidity and mortality: antibiotics for infections, stenting, prophylactic beta-blockers for portal hypertension, and endoscopic banding, sclerotherapy, or shunting for variceal bleeding. There are currently no guidelines or randomized trials on various treatment modalities for CD or CS. Hence treatment is tailored to individual patients and is dependent on their clinical presentation, extent, and site of disease [4-6].

Surgical interventions are dependent upon the risk of neoplasia - expressed as the gradual rise in tumor markers (alpha-fetoprotein and CA 19-9) and the extent of disease - expressed in the MRCP findings [5,18]. The method of choice whenever feasible for CS is liver resection. Localized forms or wide ducts confined to one lobe of the liver need hemi-hepatectomy/lobectomy [19]. First intention hepatic resection is ideal in single-lobe CD/CS because it cures all hepatic lesions with zero mortality. Associated choledochal cysts are resected at the same time if present [4,18,19]. Diffuse forms or wide ducts throughout the liver need conservative management, including antibiotics for prevention of cholangitis, endoscopic therapies, internal biliary bypass procedures, or a liver transplant [19].

The only cure for refractory and/or chronic cholangitis, malignancy, or liver failure is liver transplantation [7,8,18,19]. However, according to some studies, there was a poor prognosis after liver transplant in CS, according to a recent multicenter study, results were encouraging in terms of postoperative mortality and five-year overall survival. This led to the conclusion that the early diagnosis and timely recognition of indications for surgical intervention are of major significance [2,18]. In cases of concomitant renal failure due to dysplastic kidneys, liver plus renal transplantation may be the only curative option available [6].

Even after surgical intervention, the risks of neoplastic conversion and recurrence are not completely eliminated, so an annual follow-up is advised [18]. Surveillance is done by regular abdominal USGs, complete blood counts, liver function tests, tumor markers (CA 19-9 and CEA), and MRCP (for cholangiocarcinoma screening). Familial investigations and genetic counseling are also advised during routine follow-up [5].

## Conclusions

This case highlights the importance of high suspicion of CS in adolescent patients presenting with variceal bleeding without obvious and florid hepatic cirrhosis, particularly in resource-limited settings. Regular follow-up to assess liver and kidney functions is essential to determine the treatment and decide upon the need for either a liver transplant or a combined liver and kidney transplant.
